# Functional genomics of seed dormancy in wheat: advances and prospects

**DOI:** 10.3389/fpls.2014.00458

**Published:** 2014-09-15

**Authors:** Feng Gao, Belay T. Ayele

**Affiliations:** Department of Plant Science, University of ManitobaWinnipeg, MB, Canada

**Keywords:** dormancy, epigenetics, germination, oxidation, plant hormones, preharvest sprouting, seed, wheat

## Abstract

Seed dormancy is a mechanism underlying the inability of viable seeds to germinate under optimal environmental conditions. To achieve rapid and uniform germination, wheat and other cereal crops have been selected against dormancy. As a result, most of the modern commercial cultivars have low level of seed dormancy and are susceptible to preharvest sprouting when wet and moist conditions occur prior to harvest. As it causes substantial loss in grain yield and quality, preharvest sprouting is an ever-present major constraint to the production of wheat. The significance of the problem emphasizes the need to incorporate an intermediate level of dormancy into elite wheat cultivars, and this requires detailed dissection of the mechanisms underlying the regulation of seed dormancy and preharvest sprouting. Seed dormancy research in wheat often involves after-ripening, a period of dry storage during which seeds lose dormancy, or comparative analysis of seeds derived from dormant and non-dormant cultivars. The increasing development in wheat genomic resources along with the application of transcriptomics, proteomics, and metabolomics approaches in studying wheat seed dormancy have extended our knowledge of the mechanisms acting at transcriptional and post-transcriptional levels. Recent progresses indicate that some of the molecular mechanisms are associated with hormonal pathways, epigenetic regulations, targeted oxidative modifications of seed mRNAs and proteins, redox regulation of seed protein thiols, and modulation of translational activities. Given that preharvest sprouting is closely associated with seed dormancy, these findings will significantly contribute to the designing of efficient strategies for breeding preharvest sprouting tolerant wheat.

## INTRODUCTION

**FIGURE 1:**
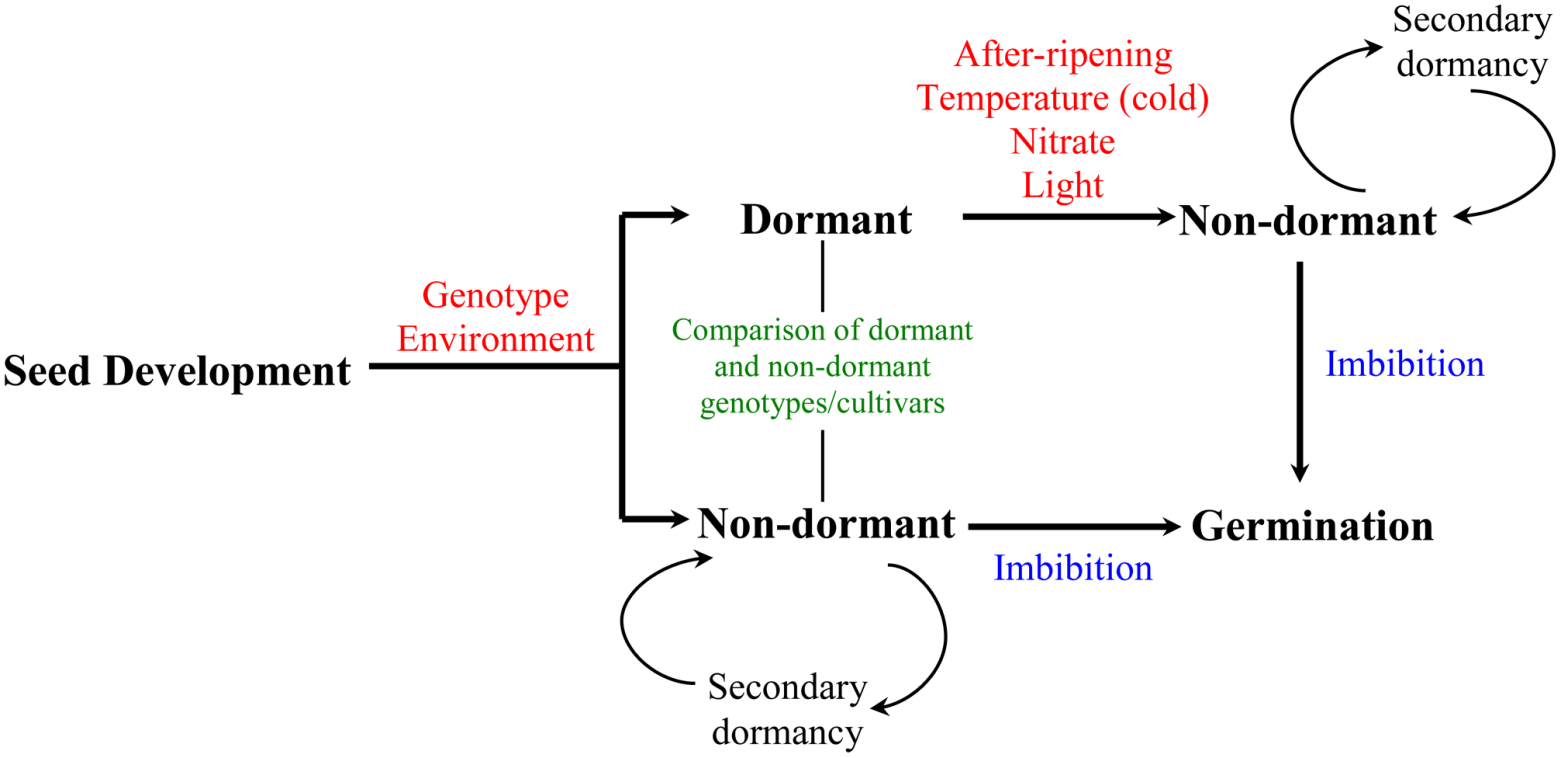
**Developmental timeline of dormancy induction and decay in seeds.** Induction and maintenance of primary dormancy during seed development is influenced by both genetic and environmental factors. Transition of mature seeds from dormant to non-dormant state can be induced by environmental signals including temperature (cold), nitrate and light, and after-ripening, a period of dry storage during which dormancy breaks down. Non-dormant seeds complete germination once imbibed or may enter secondary dormancy if the environmental conditions are unfavorable for germination. The pathway for preharvest sprouting is represented by the seeds that go directly from development-maturation to non-dormancy to germination. Seed dormancy studies in wheat mainly involve after-ripening and comparative analysis of seeds from dormant and non-dormant cultivars.

Seed is an important part of crop’s life cycle as it establishes the next generation. Its function as a basic propagation unit is influenced by three critical phases: development, dormancy, and germination. Seed dormancy refers to the inability of viable seeds to germinate under apparently optimal environmental conditions. Induction and maintenance of dormancy during seed maturation is influenced by genetic and environmental factors. Thus, seeds derived from different genotypes exhibit varying degree of dormancy at maturity; however, this can be modulated by the environment experienced by the mother plant during the seed development–maturation phase (Benech-Arnold et al., 2013). Some environmental factors appear to have similar effects on the dormancy of seeds in different genotypes; for example, high temperatures, short days, drought, and nutrient availability during seed development are generally associated with low level of seed dormancy at maturity (Rodriguez et al., 2011). Seeds can be released from the state of dormancy by exposure to a number of environmental cues including cold temperature, nitrate, and light; and also by after-ripening, which refers to a period of dry storage (Bewley and Black, 1994; **Figure 1**). Under conditions that are not favorable for germination, non-dormant seeds may re-enter dormancy, which is referred as secondary dormancy (Kermode, 2005). The effect of after-ripening in relieving seed dormancy has been shown to be associated with physiological changes that represent a critical control point in determining seeds’ capacity to germinate upon imbibition; these changes can be measured while the seeds are still in dry state and during imbibition by comparing after-ripened seeds with their dormant counterparts (Holdsworth et al., 2008a,b).

Gene transcripts stored in dry mature seeds represent residuals of mRNAs from seed developmental processes that will serve as substrates for the synthesis of proteins during imbibition (Rajjou et al., 2004; Kimura and Nambara, 2010). For example, 38% of the mRNAs represented on the GeneChip Wheat Genome Array are found to be stored in mature dormant seeds of wheat (Gao et al., 2012). Proteomic studies have shown that storage and non-storage proteins that will serve as a primary source of reduced nitrogen and participate in several cellular processes during germination, respectively, are also stored in mature dormant seeds (Bykova et al., 2011b; Gao et al., 2013). Dry after-ripening induces accumulation of reactive oxygen species (ROS), and thereby oxidative modifications of seed stored mRNAs and proteins, which upon imbibition affect their translatability and functionality, respectively (Oracz et al., 2007; El-Maarouf-Bouteau et al., 2013; Gao et al., 2013). Comparison of embryos derived from dry dormant and dry after-ripened sunflower seeds revealed that active metabolic reactions such as gene transcription appear not to occur during dry after-ripening (Meimoun et al., 2014).

Several studies that compared imbibed after-ripened and dormant seeds have shown the role of after-ripening in inducing imbibition mediated transcriptional changes that represent various biological processes including the metabolism and signaling of plant hormones, which lead to changes in seed hormone level and/or sensitivity (Preston et al., 2009; Liu et al., 2013), and epigenetic regulation of gene transcription (Nakabayashi et al., 2005; Liu et al., 2007; Bouyer et al., 2011). Furthermore, the role of after-ripening in breaking seed dormancy has been shown to be associated with imbibition mediated mechanisms operating at post-transcriptional levels, including oxidative protein carbonylation (Job et al., 2005; Oracz et al., 2007), redox regulation of seed protein thiols (Bykova et al., 2011a), and changes in seed proteome (Chibani et al., 2006; Gao et al., 2013). The physiological changes induced by after-ripening in both dry and imbibed states can take place in different seed tissues. For example, comparison of after-ripening mediated transcriptomic changes in imbibing embryos and whole seed tissues of wheat revealed that 64% of the genes regulated by after-ripening are shared by the two tissues (Bassel et al., 2011; Gao et al., 2012), and this may suggest that at least some of the changes in gene expression occur in tissues other than the embryo, such as the endosperm and aleurone fractions.

Wheat is one of the most economically important cereal crops in the world; however, its production is affected by a multitude of biotic and abiotic factors including the occurrence of wet and moist conditions prior to harvest that causes preharvest sprouting, which refers to the germination of mature seeds on the mother plant. Preharvest sprouting in cereals causes substantial yield, crop grade, and end-use quality losses; and the total worldwide direct financial loss associated with field sprouting is estimated to be ∼$1 billion annually (Black et al., 2006). The degree of tolerance/susceptibility of wheat seeds to preharvest sprouting is closely associated with the level of dormancy manifested in the seed. While excessive seed dormancy causes delayed germination and poor stand establishment (Derera, 1989), reduced dormancy is also undesirable in the production of cereal crops such as wheat as it makes the seeds susceptible to preharvest sprouting. The presence of moderate level of dormancy is, therefore, desirable to prevent seed sprouting prior to harvest. However, the domestication and breeding programs of cereal crops including wheat have been aimed at selection against seed dormancy so as to achieve quick and uniform germination (Simpson, 1990). As the result of this selective pressure, most of the commercial wheat cultivars are prone to preharvest sprouting. Elucidation of the molecular mechanisms of seed dormancy in wheat is critical to develop cultivars with enhanced tolerance to preharvest sprouting. Recent advances in wheat genomic data generation, assembly, and annotation along with the application of functional genomics approaches such as transcriptomics, proteomics, and metabolomics have extended our knowledge with this respect. This review highlights recent progresses in our understanding of the molecular switches in the transcriptional and post-transcriptional programs associated with the control of seed dormancy and preharvest sprouting in wheat.

## HORMONAL REGULATION OF WHEAT SEED DORMANCY

The role of plant hormones in regulating seed dormancy and germination through a variety of synergistic and antagonistic interactions is well described (Kucera et al., 2005; Finkelstein et al., 2008; Linkies and Leubner-Metzger, 2012). Although prime attention has been given in unraveling the molecular mechanisms underlying the functions of abscisic acid (ABA) and gibberellin (GA) in the control of seed dormancy, numerous recent studies have provided important insights into the molecular features underlying the role of other plant hormones such as jasmonate, brassinosteroid, and ethylene in the regulation of seed dormancy and germination. As most of these studies have been focused on the seeds of dicot species, understanding of this phenomenon in the seeds of cereal crops, specifically wheat, has been lagging. However, recent improvements in bioinformatics for sequence-based identification of candidate wheat hormonal ortholog genes from publicly available databases along with the use of functional genomics approaches in studying hormonal regulation of wheat seed dormancy and germination is advancing our knowledge of the underlying molecular mechanisms.

### MOLECULAR SWITCHES ASSOCIATED WITH ABSCISIC ACID METABOLISM

Abscisic acid is a major player in regulating seed dormancy (Rodriguez-Gacio et al., 2009), and its level in seeds is controlled by a balance between its biosynthesis and inactivation (Nambara et al., 2010). While its biosynthesis is catalyzed by several enzymes, NINE-*CIS*-EPOXYCAROTENOID DIOXYGENASE (NCED) appears to be the major regulator of ABA synthesis during seed maturation. In *Arabidopsis*, *NCED6* and *NCED9* are highly expressed in developing seeds, and mutational analysis of these two genes indicated their role in inducing ABA synthesis and seed dormancy (Lefebvre et al., 2006). Developing seeds of barley and wheat accumulate ABA in which the level reaches a maximum during the mid- and late-maturation phases (Suzuki et al., 2000; Chono et al., 2006), suggesting the significance of ABA for the induction of embryo dormancy (Garello and Le Page-Degivry, 1999). *NCED2* of barley is suggested to have a primary role in regulating ABA level during seed development (Chono et al., 2006).

Inactivation of the biologically active ABA takes place through hydroxylation or conjugation with sugars. ABA hydroxylation at C-8′ position is considered to be the predominant ABA inactivation pathway (Nambara and Marion-Poll, 2005), and it appears to be critical in regulating seed dormancy release (Gubler et al., 2005). This reaction is catalyzed by ABA 8′-hydroxylase (ABA8′OH), a cytochrome P450 monooxygenase that is encoded by the *ABA8′OH* (*CYP707A*) gene family (Nambara et al., 2010). Previous studies in barley have shown that after-ripening of dormant seeds activates the transcription of *ABA′OH1* in embryos during imbibition, and this is associated with a decline in ABA level (Millar et al., 2006; Gubler et al., 2008). RNAi based mutational analysis of *ABA′OH1* revealed the critical role of this gene in controlling embryo ABA content and dormancy release in barley (Gubler et al., 2008). The embryonic ABA content is also shown to be positively correlated with the level of dormancy in sorghum seeds (Benech-Arnold et al., 1999). However, studies in wheat have reported no association between the levels of dormancy and embryo ABA (Walker-Simmons, 1987; Morris et al., 1989; King, 1993). In agreement with these reports, a recent transcriptomic and targeted metabolic analysis between whole dormant and after-ripened wheat seeds showed a difference neither in the expression of ABA metabolic genes nor in seed ABA content in both dry and hydrated states (Liu et al., 2013). Seed ABA level, however, declined similarly during imbibition in both dormant and after-ripened seed samples, which was accompanied by transcriptional repression and activation of specific wheat orthologs of *NCED* and *ABA′OH* genes, respectively. Contrary to this, a recent study showed that embryos of a wheat double mutant of *ABA′OH1-A* and *ABA′OH1-D* contain higher amounts of ABA during seed development, and exhibit lower germination than those derived from the corresponding wild type (Chono et al., 2013), implicating the role of ABA catabolism in the regulation of seed dormancy and germination in wheat. These results emphasize the need of further studies such as functional analysis of the associated molecular elements in order to gain important insights into the involvement of ABA metabolism in the regulation of wheat seed dormancy and germination.

### MOLECULAR SWITCHES ASSOCIATED WITH ABSCISIC ACID SIGNALING

**FIGURE 2:**
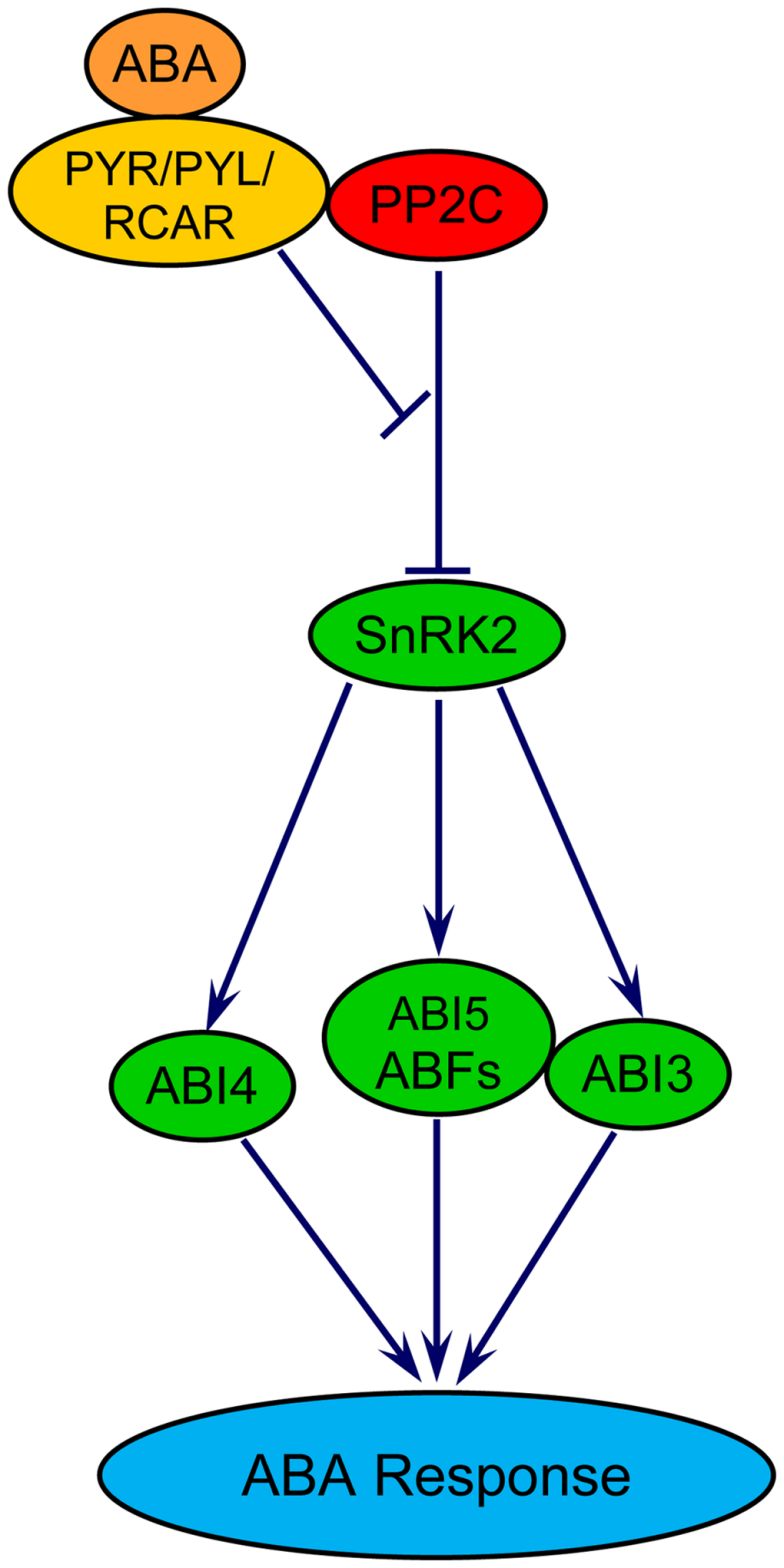
**Model for ABA signaling in plants.** PYR/PYL/RCAR, pyrabactin resistance/PYR-like/regulatory components of ABA receptors; PP2C, protein phosphatase 2C; SnRK, SNF1-related protein kinase2; ABF, ABA responsive element binding factor; ABI3/4/5, ABA insensitive 3/4/5. ABI3 and ABI5 proteins physically interact with each other.

The role of ABA in delaying wheat seed germination has been demonstrated (Liu et al., 2013; Chitnis et al., 2014). This effect of ABA has been shown to be associated with transcriptional repressions of biological processes related to chromatin assembly, cytoplasmic membrane-bound vesicle, and carbohydrate metabolism such as starch and maltose degradation, and cell wall hydrolysis; and transcriptional activation of GA catabolism (Liu et al., 2013). Central to ABA signaling in seeds are three core components: PYRABACTIN RESISTANCE/PYRABACTIN-LIKE/REGULATORY COMPONENTS OF ABA RECEPTORS (PYR/PYL/RCAR), PROTEIN PHOSPHATASE 2Cs (PP2Cs) and SNF1-RELATED PROTEIN KINASE 2s (SnRK2s; reviewed in Nambara et al., 2010; **Figure 2**). The binding of ABA to its receptor PYR/PYL/RCAR forms a complex, which in turn interacts with and inhibits the activity of PP2Cs that negatively regulate ABA signaling through repression of SnRK2s, the positive regulators of downstream targets. Inhibition of the PP2Cs leads to de-repression of SnRK2s, which phosphorylate and activate downstream transcription factors including the bZIP-type transcription factors ABFs and ABSCISIC ACID INSENSITIVE5 (ABI5), the AP2-type transcription factor ABI4, and the B3-type protein ABI3 that are key to regulate the expression of ABA responsive genes in seeds (Nambara et al., 2010). When there is no ABA, PP2Cs dephosphorylate and deactivate SnRK2s. The ABA signaling pathway involving these molecular components appear to be conserved in the seeds of both dicot and monocot species (Kim et al., 2012).

Analyses of mutational effects and QTL in wheat have demonstrated the significance of seed ABA sensitivity in regulating dormancy (Kawakami et al., 1997; Noda et al., 2002; Schramm et al., 2010, 2012, 2013). Indeed, dormant wheat seeds exhibit increased sensitivity to ABA as compared to the non-dormant counterparts (Walker-Simmons, 1987; Morris et al., 1989; Corbineau et al., 2000). A study in rice has demonstrated that seeds expressing the ABA receptor ortholog, *OsPYL/RCAR5*, are hypersensitive to ABA during germination (Kim et al., 2012). A recent comparative transcriptomic analysis of the whole seed (consisting of the embryo, endosperm, aleurone layer, and testa) between dormant and after-ripened samples of wheat, however, revealed no differential transcription of wheat PYR/PYL/RCAR orthologs represented in the GeneChip Wheat Genome Array during imbibition (Liu et al., 2013). This finding might suggest that the specific orthologs identified in this study do not participate in the activation of ABA signaling or they are subjected to regulations by post-transcriptional mechanisms. Thus, further characterization of wheat PYRPYL/RCAR orthologs in seeds is necessary to gain insights into their functionality with respect to dormancy and the associated regulatory mechanisms.

On the contrary, coordinated transcriptional repression of specific wheat orthologs of *SnRK2* and *ABI5* was apparent in after-ripened seeds (Liu et al., 2013). A member of wheat SnRK2, PKABA1 (Gomez-Cadenas et al., 1999), interacts with a seed specific wheat homolog of ABI5, TaABF1 (Johnson et al., 2002), thereby activating the transcription of ABA responsive genes (Hobo et al., 1999). Although the physiological functions of PKABA1 and TaABF1 remains to be elucidated, the association of their transcriptional repression in the embryo with reduced seed dormancy and sensitivity to ABA in wheat and other cereal seeds might highlight the significance of these ABA signaling components in the control of seed dormancy and germination (Rodriguez et al., 2009; Rikiishi and Maekawa, 2010). It is well established that the ABI3 protein acts upstream of ABI5 in the ABA signal transduction pathway, and thereby regulates its action to execute ABA-dependent growth inhibition during germination (Lopez-Molina et al., 2002). Consistent with this, the expression of *ABI3* ortholog of wheat, *VIVIPAROUS1* (*VP1*) in the embryo correlates positively with the degree of seed dormancy and sensitivity to ABA (Nakamura and Toyama, 2001; Laethauwer et al., 2012). Furthermore, developing embryos of wheat seeds expressing the *VP1* ortholog of oat exhibit increased sensitivity to ABA (McKibbin et al., 2002). In contrast to these results, analysis of the whole imbibing dormant and after-ripened seeds of wheat exhibit no apparent differential transcription of *VP1* (Liu et al., 2013), which might be attributed to the misplicing nature of wheat *VP1* that compromises its expression (McKibbin et al., 2002).

Other ABA signaling components that are implicated to have roles in seed dormancy include the S-PHASE KINASE-ASSOCIATED PROTEIN1 (SKP1), ABI3-INTERACTING PROTEIN2 (AIP2), and LIPID PHOSPHATE PHOSPHATASE2 (LPP2). The SKP1 protein forms a subunit of the SCF complex E3 ligase and regulates ABA signaling through degradation of specific proteins (Sadanandom et al., 2012). Since overexpression of the wheat *SKP1-like1* (*TSK1*) in *Arabidopsis* causes delay in seed germination and hypersensitivity to ABA, it has been suggested that the PP2Cs, which act as a negative regulator of ABA signaling, might be the targets of the SCF complex formed by SKP1 (Li et al., 2012).

The AIP2 is an E3 ligase that represses ABA signaling by targeting ABI3 for degradation, and null mutation in the *AIP2* of *Arabidopsis* leads to enhanced seed sensitivity to ABA (Zhang et al., 2005). Comparative transcriptomic analysis of dormant and after-ripened whole wheat seeds in both dry and hydrated states, however, showed no differential transcription or downregulation of the *SKP1* and *AIP2* orthologs in the after-ripened seeds (Liu et al., 2013). This might imply that the role of *SKP1* and *AIP2* in seed dormancy is likely mediated by post-transcriptional regulation.

The LPP2 catalyzes the synthesis of phosphatidic acid, which is involved in ABA signaling in germinating *Arabidopsis* seeds (Katagiri et al., 2005). It has been shown through mutational study that LPP2 functions as a negative regulator of ABA signaling upstream of ABI4, one of the transcription factors that mediate ABA-induced gene transcription. Transcriptional activations of specific *LPP* orthologs in the coleorhiza and at whole-seed level following after-ripening of dormant barley and wheat seeds, respectively, might suggest the significance of LPP in regulating ABA sensitivity and dormancy in cereal seeds (Barrero et al., 2009; Liu et al., 2013).

In addition, Xi et al. (2010) identified *MOTHER OF FT AND TFL1* (*MFT*) as a molecular element capable of enhancing *Arabidopsis* seed germination by conferring negative feedback regulation of ABA signaling, which is mediated via transcriptional repression of *ABI5*. However, the wheat homolog of *MFT* appears to have an opposite role as its expression in the embryo is positively correlated with the level of seed dormancy (Nakamura et al., 2011). Indeed, *MFT* is repressed in the whole seed tissue of imbibing after-ripened relative to the corresponding dormant wheat samples (Liu et al., 2013). However, the mechanisms by which MFT regulates dormancy in wheat seeds remain to be elucidated.

On the contrary to the findings of other studies that suggested a relationship between dormancy and seed sensitivity to ABA, a study by Gerjets et al. (2010) showed a clear correlation between the rate of seed after-ripening and susceptibility to sprouting but with no direct relationship between after-ripening and embryo ABA responsiveness. Whether wheat seed dormancy and after-ripening are controlled by separate genetic pathways, as shown in *Arabidopsis* (Carrera et al., 2008), awaits further investigation.

### MOLECULAR FEATURES RELATED TO GIBBERELLIN METABOLISM AND SIGNALING

The role for GA in the control of seed dormancy and germination has been described (Finch-Savage and Leubner-Metzger, 2006). The amount of bioactive GAs in plant tissues is controlled by the balance between their synthesis and inactivation, which are mainly regulated by genes encoding GA 20-OXIDASE (*GA20ox*) and GA 3-OXIDASE (*GA3ox*), and GA 2-OXIDASE (*GA20ox*), respectively (Yamaguchi, 2008). Differential transcriptions of the orthologs of these genes in response to after-ripening or between seeds derived from dormant and non-dormant cultivars of cereal crops have implicated GA in the regulation of dormancy and germination in these species. For example, imbibition mediated transcriptional activation of *GA20ox* and *GA3ox* orthologs occurs in the embryo and whole seed of after-ripened as compared to dormant samples of barley and wheat, respectively (Gubler et al., 2008; Liu et al., 2013). Furthermore, transcriptional induction of specific *GA20ox* orthologs accompanies increased bioactive GA_4_ level in the embryos of non-dormant seeds of sorghum, while those derived from dormant seeds exhibit upregulation of specific *GA2ox* orthologs and low level of GA_4_ (Rodriguez et al., 2012). Mutational analysis of these genes will have a significant contribution in elucidating the molecular mechanisms that underlie the regulation of wheat seed dormancy and germination by GA.

The GA signal in plants is perceived by a soluble receptor protein, GIBBERELLIN INSENSITIVE DWARF1 (GID1), which was first identified in rice (Ueguchi-Tanaka et al., 2005). The orthologs of GID1 have also been identified in barley (Chandler et al., 2008) and wheat (Li et al., 2013). Although the function of the wheat ortholog of GID1 with respect to seed dormancy awaits characterization, mutation in *GID1* of rice has been shown to lead to the repression of α-amylase synthesis with no apparent inhibitory effect on germination (Ueguchi-Tanaka et al., 2005).

Another key component of GA signaling in plants is the DELLA protein, which acts as repressor of GA activated responses; and it is degraded by GA through ubiquitination (Sun, 2011). *Arabidopsis* consists of five DELLAs including GA INSENSITIVE (GAI), REPRESSOR OF GA1-3 (RGA), RGA-LIKE1 (RGL1), RGL2, and RGL3 (Davière and Achard, 2013), of which RGL2 is considered the major repressor of seed germination (Lee et al., 2002; Tyler et al., 2004). The DELLA proteins of cereals such as SLENDER RICE1 (SLR1) of rice (Ikeda et al., 2001); SLENDER1 (SLN1) of barley (Chandler et al., 2002) and REDUCED HEIGHT (RHT) of wheat (Peng et al., 1999) are encoded by a single gene. The seeds of DELLA mutants of barley are non-dormant and show enhanced activity of α-amylase in the aleurone layer (Chandler, 1988).

Apart from DELLA proteins, GAMYB, a GA-regulated MYB transcriptional regulator, plays an important role in GA signaling in cereal aleurone cells (Gubler et al., 1995, 1999). By binding directly to the GA-response element in the promoter regions, GAMYB mediates GA regulated transcriptional activation of hydrolytic enzymes, in particular that of α-amylase, in cereal aleurone (Gubler et al., 1995, 1999). Consistently, mutation in the rice ortholog of *GAMYB* leads to repression of α-amylase genes with no effect on germination (Kaneko et al., 2004). The function of GAMYB protein in cereal aleurone is repressed by *KINASE ASSOCIATED WITH GAMYB1* (*KGM1*), and this may contribute to the inhibition of expression of hydrolase genes (Woodger et al., 2003).

A recent whole-seed based transcriptomic study showed no differential transcription of wheat orthologs of *GID1*, *RHT*, *GAMYB*, and *KGM* represented in the GeneChip Wheat Genome Array between dormant and after-ripened seeds, although the transcription of GA responsive genes such as those encoding amylases and cell wall hydrolases is induced in response to after-ripening (Liu et al., 2013). These results might suggest that wheat seed responsiveness to GA is regulated by post-transcriptional mechanisms or operate independent of these GA signaling factors. For example, a study by Cao et al. (2006) suggested the presence of DELLA-independent GA signaling pathway in germinating *Arabidopsis* seeds.

### MOLECULAR SWITCHES RELATED TO OTHER PLANT HORMONES

Previous studies implicate jasmonate, ethylene, and brassinosteroid (BR) in the regulation of seed dormancy (Kucera et al., 2005; Matilla and Matilla-Vázquez, 2008; Linkies and Leubner-Metzger, 2012). Transcriptomic analysis of dormancy at whole-seed level in wheat has revealed imbibition induced changes in the expression of specific orthologs of genes related to jasmonate, ethylene, and BR in response to after-ripening (Liu et al., 2013; Chitnis et al., 2014), providing insights into the roles of these hormones in the regulation of wheat seed dormancy and germination. With respect to jasmonates, transcriptional activation of specific orthologs of the jasmonate biosynthetic genes, *ALLENE OXIDE SYNTHASE* (*AOS*), *3-KETOACYL COENZYME A THIOLASE3* (*KAT3*), and *LIPOXYGENASE5* (*LOX5*), was observed during imbibition of after-ripened seeds. This along with the presence of higher amount of jasmonate-isoleucine in imbibed after-ripened than dormant seeds might imply the role of jasmonate in wheat dormancy control. Consistently, a recent report by Jacobsen et al. (2013) indicated that methyl jasmonate reduces the level of seed dormancy in wheat. This role of methyl jasmonates has been shown to be mediated by changes in embryo ABA content and the expressions of *NCED1* and *ABA8′OH1* genes. Furthermore, after-ripening leads to the repression of specific wheat orthologs of *MITOGEN ACTIVATED PROTEIN KINASE1* (*MAPK1*), which acts as a negative regulator of JA signaling and the transcriptional activation of JA responsive genes during seed imbibition (Liu et al., 2013). Similarly, the orthologs of jasmonate biosynthesis genes, *JASMONATE 12-OXOPHYTODIENOIC ACID REDUCTASE* (*OPR*) and *AOS*, and jasmonate receptor gene, *CORONATINE INSENSITIVE1* (*COI1*), exhibited transcriptional activation in the coleorhiza of after-ripened barley seeds (Barrero et al., 2009).

Using a whole-seed system, it has been shown recently that after-ripening of dormant wheat seeds leads to imbibition mediated transcriptional activation of specific orthologs of BR biosynthetic genes, *DE-ETIOLATED2* (*DET2*) and *DWARF4* (*DWF4*; Chitnis et al., 2014). Furthermore, upregulation of orthologs of *BR SIGNALING KINASE* (*BSK*), which functions as a positive regulator of BR signaling (Tang et al., 2008); and downregulation of specific orthologs of *BR INSENSITIVE2* (*BIN2*) that functions as a negative regulator of BR signaling (Li and Nam, 2002) were apparent during imbibition of after-ripened seeds. These transcriptional regulations of the BR biosynthesis and signaling components during imbibition of after-ripened wheat seeds have been shown to be coordinated with the transcriptional induction of BR responsive orthologs including *PACLOBUTRAZOL RESISTANCE* (*PRE*) and *BR ENHANCED EXPRESSION* (*BEE*; Chitnis et al., 2014), which are involved in cell elongation (Friedrichsen et al., 2002; Zhang et al., 2009), a process necessary for the completion of seed germination. These results altogether imply the role of BR in the control of wheat seed dormancy and germination.

Previous studies in *Arabidopsis* indicated that BR regulates dormancy by counteracting the effect of ABA (Steber and McCourt, 2001; Divi and Krishna, 2010) and enhancing the production of ethylene, which has been implicated in the regulation of seed dormancy in monocot species such as wild oat (Adkins and Ross, 1981). The role of BR in inducing ethylene synthesis in *Arabidopsis* appears to be post-transcriptional, by mediating the stabilization of the ethylene biosynthetic enzyme 1-AMINOCYCLOPROPANE-1-CARBOXYLIC ACID (ACC) SYNTHASE (ACS) that catalyzes the first committed and rate limiting step (Hansen et al., 2009). However, if the same mechanisms underlie the role of BR in the control of wheat dormancy remains to be elucidated. Apart from ACS, the synthesis of ethylene is catalyzed by ACC OXIDASE (ACO). Wheat orthologs of *ACO* exhibit upregulation at the whole-seed level during imbibition of after-ripened as compared to dormant samples (Chitnis et al., 2014). This along with the transcriptional induction of wheat orthologs of the ethylene receptor, *ETHYLENE RESPONSE SENSOR1* (*ERS1*), and ethylene regulated genes in response to after-ripening implicate ethylene in the control of wheat seed dormancy and germination. Contrary to this, in other cereal crops such as barley and red rice, the role of ethylene has been associated with the promotion of germination of non-dormant seeds but not with dormancy loss (Locke et al., 2000; Gianinetti et al., 2007). Overall, the role of ethylene in regulating seed dormancy appears to be less obvious at this point (Matilla and Matilla-Vázquez, 2008), emphasizing the need of further studies to investigate if it functions as a regulator of seed dormancy in different plant species.

## REGULATION OF WHEAT SEED DORMANCY BY EPIGENETIC MECHANISMS

Apart from transcriptional regulatory events, epigenetic mechanisms including histone modifications, DNA methylation, and chromatin remodeling regulate gene expression in plant developmental processes (Cooke et al., 2012). Previous studies in *Arabidopsis* highlight the significance of these epigenetic mechanisms in the control of seed dormancy (Graeber et al., 2012). Evidences suggesting the role for epigenetic regulation of gene expression in the control of dormancy and germination in cereal seeds are emerging. For example, genes linked to chromatin structure and histone genes are found to be overrepresented among genes transcriptionally induced at whole-seed level during germination of non-dormant barley seeds, specifically during the late germination phase (An and Lin, 2011). Furthermore, consistent expression of the SET family transcription factors that play a role in histone methylation (Malagnac et al., 2002; Xiao et al., 2003) is apparent in the embryos during germination of non-dormant rice seeds (Howell et al., 2009). In accordance, wheat orthologs transcriptionally activated in the whole seed during imbibition of after-ripened samples are enriched in the chromatin assembly gene ontology (Gao et al., 2012). These orthologs include those representing histone proteins such as H4, HTA11, HTA12, HTB11, HTB9, and FASCIATA1, a histone binding protein, that are important for nucleosome and chromatin formation, and thereby regulation of gene expression. Furthermore, orthologs of histone modification genes including *CHROMOMETHYLASE3* and *METHYLTRANSFERASE1* exhibit transcriptional induction in imbibed after-ripened relative to dormant seeds. These results suggest the role of epigenetic regulation of gene expression in mediating after-ripening induced developmental switch of wheat seeds from dormant to non-dormant state. Further studies are required to identify more dormancy related epigenetic regulators and define how the orthologs related to epigenetic mechanisms are involved in the control of wheat seed dormancy and germination.

## REGULATION OF WHEAT SEED DORMANCY BY OXIDATIVE MODIFICATION OF GENE TRANSCRIPTS AND PROTEINS

Seed dormancy release by dry after-ripening is associated with autooxidation-mediated accumulation of ROS such as superoxide, hydrogen peroxide, and hydroxyl radicals (El-Maarouf-Bouteau and Bailly, 2008). Apart from playing signaling roles in several cellular processes, the ROS produced during dry after-ripening are involved in the non-enzymatic oxidation of selected seed stored mRNAs and proteins, which lead to decreased protein synthesis and impaired protein function upon imbibition, respectively (El-Maarouf-Bouteau et al., 2013). The first line of evidence for targeted oxidation of such seed stored transcripts during after-ripening and its association with dormancy release has come from the study of sunflower embryos (Bazin et al., 2011). A subsequent study in wheat using a whole-seed system confirmed that oxidative modification of specific seed stored mRNAs occurs during dry after-ripening (Gao et al., 2013). The oxidized transcripts in wheat seeds correspond to orthologs over-represented in nutrient reservoir activity, such as those encoding seed storage proteins gliadin and glutenin, and α-amylase inhibitor activity, such as those encoding the α-amylase/trypsin inhibitor designated as CM (because of its solubility in chloroform/methanol). Dry after-ripening also induces oxidation of other specific transcripts corresponding to granule bound starch synthase I (GBSSI), peroxidase (POX), and ribosomal protein. These results indicate the significance of post-transcriptional regulation of the associated biological processes in the control of dormancy in wheat seeds. Therefore, it is plausible to suggest that targeted oxidation of seed stored mRNA is one of the conserved mechanisms underlying the regulation of seed dormancy in both monocot and dicot species. Another mechanism by which the ROS regulates seed dormancy and germination is by inducing modifications to the redox state of seed protein thiols, which ultimately lead to changes in protein properties and functions (Buchanan and Balmer, 2005). Using a whole-seed based proteomic analysis, Bykova et al. (2011a) showed that after-ripening mediated seed dormancy release in wheat is associated with changes in the thiol-redox state of proteins involved in carbohydrate metabolism, synthesis of secondary metabolites, energy and amino acid metabolism, genetic information processing, transport, and antioxidative defense. Differential redox state of seed proteins is also apparent between wheat seeds derived from dormant and non-dormant hybrid genotypes (Bykova et al., 2011b). The role of ROS in regulating cellular processes is also mediated by their interaction with ABA and GA signaling (El-Maarouf-Bouteau and Bailly, 2008). Consistently, changes in the redox state of wheat seed proteins appear to be modulated by GA and ABA (Bykova et al., 2011a), although the underlying mechanisms remain to be investigated.

## WHEAT SEED DORMANCY AND CHANGES IN SEED PROTEOME

In addition to induction of changes in protein redox status, dry after-ripening triggers differential abundance of specific seed stored proteins in the whole seed of wheat, including repression of those identified as storage protein triticin, antioxidative superoxide dismutase (SOD), α-amylase/trypsin inhibitor designated as CM16, a protease inhibitor cystatin, and 14-3-3 proteins (14-3-3s) that controls ABA action in seeds positively (Gao et al., 2013). The repression of these proteins is likely triggered by ROS mediated oxidation, and the results imply the association of seed dormancy release by dry after-ripening with enhanced degradation or proteolysis and hydrolysis of storage reserves, loss of seed sensitivity to ABA, and maintenance of the cellular ROS homoeostasis. While some whole-seed proteins of wheat are regulated by imbibition irrespective of seed dormancy status (Park et al., 2013), after-ripening causes the repression of specific proteins, including storage proteins triticin and globulin 3, GBSSI, protease inhibitor serpins, eukaryotic translation initiation factors (eIF) 5A1 and eIF6, and protein disulfide isomerase (PDI) during seed imbibition (Gao et al., 2013). These results suggest that changes in the translation of specific seed proteins form an integral part of the mechanisms underlying the after-ripening mediated dormancy release and subsequent germination of wheat seeds.

**FIGURE 3:**
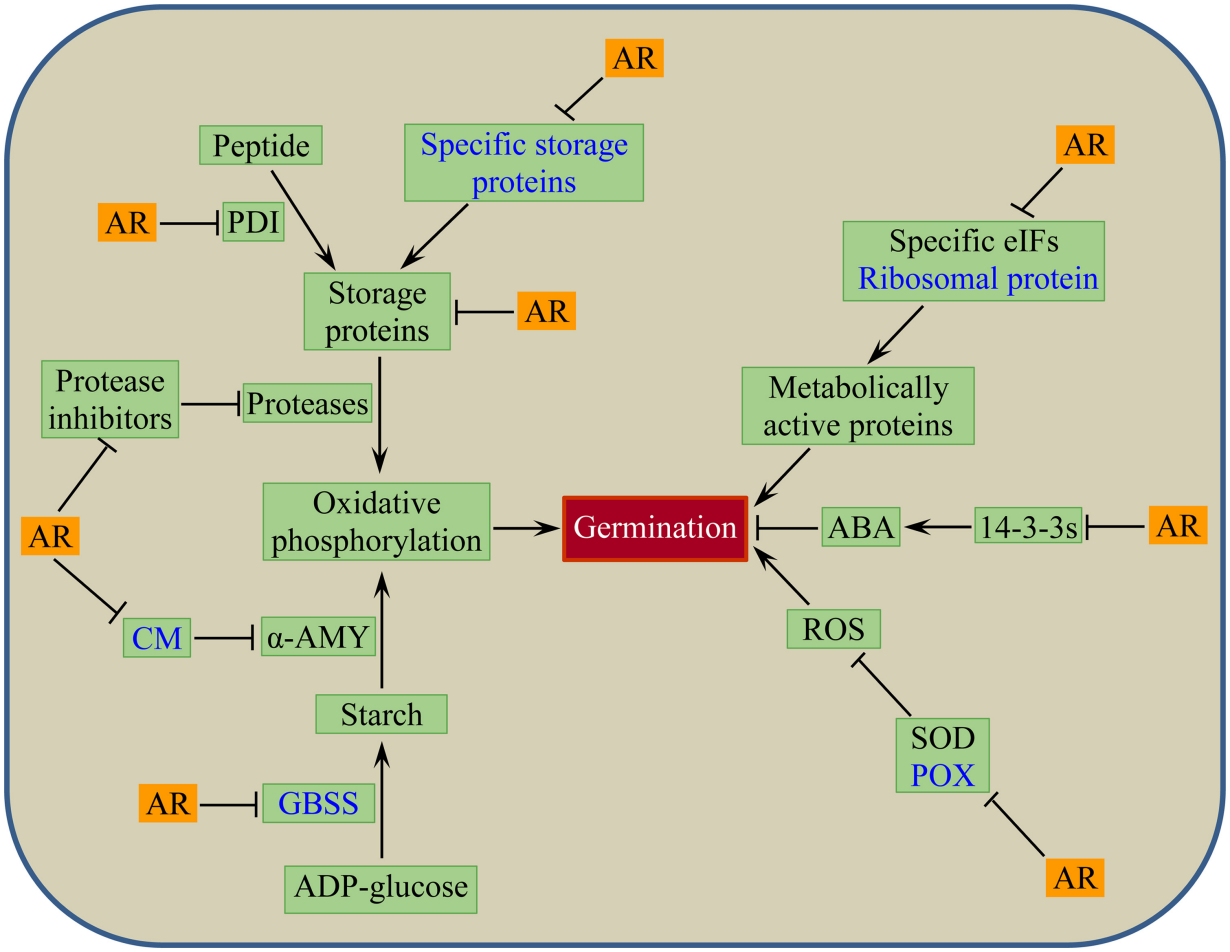
**Putative model for post-transcriptional regulation of after-ripening mediated seed dormancy release in wheat: events or components associated with after-ripening induced oxidative modification of seed stored mRNAs in dry state (shown in blue text) and changes in seed protein abundance in both dry and imbibed states.** AR, after-ripening; PDI, protein disulfide isomerase; CM, chloroform: methanol soluble α-amylase/trypsin inhibitor; α-AMY, alpha-amylase; GBSS, granule bound starch synthase; eIFs, eukaryotic translation initiation factors; 14-3-3s, 14-3-3 proteins; ABA, abscisic acid; SOD, superoxide dismutase; POX, peroxidase; ROS, reactive oxygen species.

The putative wheat seed dormancy controlling mechanism associated with after-ripening mediated oxidative modification of seed stored mRNAs and change in seed proteome is depicted by a model shown in **Figure 3**. The model postulates that dry after-ripening suppresses the synthesis of CM, GBSSI, POX, and specific seed storage proteins through oxidative modifications of the corresponding seed stored mRNAs, and thereby contributes to the release of seeds from the state of dormancy. After-ripening also enhances proteolysis and hydrolysis of storage proteins and starch by repressing the activity of proteases and amylase inhibitors, such as serpins, cystatins, and CM proteins. Furthermore, through inhibition of the activity of PDIs and starch synthases that are involved in the accumulation of storage proteins and starch, after-ripening promotes the degradation of seed reserves, producing substrates for oxidative phosphorylation to generate energy that fuels embryo growth. The model also depicts the role of after-ripening in inducing the loss of seed sensitivity to ABA through repression of the 14-3-3s that control ABA action in germinating seeds (Schoonheim et al., 2007) and accumulation of ROS via inhibition of antioxidative enzymes such as SOD. The oxidation of seed stored mRNA corresponding to ribosomal protein and repression of specific translation factors such as eIF6 and eIF5A1 in response to after-ripening implicate selective *de novo* synthesis of metabolically active proteins that are essential for dormancy decay and germination.

## CONCLUSION AND FUTURE PROSPECTS

Previous studies have shown that the role of after-ripening in enhancing dormancy breakage in wheat seeds is associated with changes in gene expression and seed proteome, and targeted oxidation of seed stored transcripts and proteins. Beyond oxidative modification, after-ripening is likely to cause other forms of RNA/protein or epigenomic modifications that potentially trigger seed dormancy decay in dry and/or imbibed states. Thus, application of functional genomic approaches for global analysis of such modifications in response to after-ripening will contribute significantly to advancing our understanding of the molecular mechanisms underlying wheat seed dormancy. Furthermore, most dormancy studies in wheat are focused on post-harvest of seeds. However, the biotic and abiotic factors experienced by the mother plant during pre- and post-dormancy induction phases of wheat seed development are critical in regulating the state of dormancy manifested by freshly harvested seeds. Therefore, global comparative studies with respect to conditions experienced during seed development are crucial to identify important wheat orthologs that control dormancy status in wheat seeds. For example, transcriptomic analysis of differentially expressed genes between wheat embryos harvested from mature seeds grown under low and high temperature regimes enabled the identification of a wheat homolog of *MFT* as an important regulator of seed dormancy (Nakamura et al., 2011).

Functional assignment of candidate wheat orthologs is mostly performed based on sequence similarity or identity. However, sequence-based functional assignment of gene orthologs may lead to incorrect annotations. This is because orthologs that are highly divergent across species may have the same function while those with similar sequences may have different functions. Furthermore, new functions of the wheat orthologs cannot be identified through such sequence-based annotations. Therefore, investigating the physiological functions of the candidate orthologs or proteins identified through “omic” approaches as regulators of wheat seed dormancy is important. As common wheat is hexaploid (2*n* = 42) that contains three subgenomes, namely A, B, and D; and seven pairs of homoeologous chromosomes per subgenome, each gene has three copies. Previous studies have shown that the genomic contributions to the total expression of a target gene vary with tissues and developmental stages (Nomura et al., 2005; Deol et al., 2013). Therefore, identifying and characterizing the homoeologs of a candidate gene from each of the three genomes, and elucidating the degree of their contributions to the total expression of a target gene are important for detailed dissection of the underlying molecular mechanisms regulating seed dormancy. Genomic resources assembled from the whole genome sequence made available by using the next-generation sequencing technology (Brenchley et al., 2012) and those being generated through the on-going chromosome-based wheat genome sequencing by the International Wheat Genome Sequencing Consortium will have a significant contribution in accelerating the identification and functional analysis of seed dormancy related genes in wheat.

In summary, the application of functional genomics technologies in studying wheat seed dormancy and germination has enhanced the discovery of transcriptional and post-transcriptional switches that form an integral part of the molecular mechanisms underlying the control of seed dormancy and germination in wheat. Since preharvest sprouting is closely associated with seed dormancy, the findings will have significant contributions in accelerating wheat breeding for improved preharvest sprouting tolerance.

## Conflict of Interest Statement

The authors declare that the research was conducted in the absence of any commercial or financial relationships that could be construed as a potential conflict of interest.
